# Effects of Taxol on Regeneration in a Rat Sciatic Nerve Transection Model

**DOI:** 10.1038/srep42280

**Published:** 2017-02-09

**Authors:** Shih-Tien Hsu, Chun-Hsu Yao, Yuan-Man Hsu, Jia-Horng Lin, Yung-Hsiang Chen, Yueh-Sheng Chen

**Affiliations:** 1 Department of Obstetrics and Gynaecology, Taichung Veterans General Hospital, Taichung, Taiwan; 2 School of Medicine, Lab of Biomaterials, Graduate Institute of Basic Medical Science, Graduate Institute of Biomedical Sciences, Graduate Institute of Integrated Medicine, Department of Biological Science and Technology, Research Center for Chinese Medicine & Acupuncture, China Medical University, Taichung, Taiwan; 3 Biomaterials Translational Research Center, China Medical University Hospital, Taichung, Taiwan; 4 Department of Bioinformatics and Medical Engineering, Department of Psychology, College of Medical and Health Science, Asia University, Taichung, Taiwan; 5 Department of Fiber and Composite Materials, Feng Chia University, Taichung, Taiwan

## Abstract

Recent studies describe taxol as a candidate treatment for promoting central nerve regeneration. However, taxol has serious side effects including peripheral neurotoxicity, and little information is known about the effect of taxol on peripheral nerve regeneration. We investigated the effects of taxol on regeneration in a rat sciatic nerve transection model. Rats were divided into four groups (n = 10): normal saline (i.p.) as the control, Cremophor EL vehicle, and 2 or 6 mg/kg of taxol in the Cremophor EL solution (four times in day-2, 4, 6, and 8), respectively. We evaluated neuronal electrophysiology, animal behaviour, neuronal connectivity, macrophage infiltration, location and expression levels of calcitonin gene-related peptide (CGRP), and expression levels of both nerve growth factors and immunoregulatory factors. In the high-dose taxol group (6 mg/kg), neuronal electrophysiological function was significantly impaired. Licking latencies were significantly changed while motor coordination was unaffected. Neuronal connectivity, macrophage density, and expression levels of CGRP was dramatically reduced. Expression levels of nerve growth factors and immunoregulatory factors was also reduced, while it was increased in the low-dose taxol group (2 mg/kg). These results indicate that taxol can modulate local inflammatory conditions, impair nerve regeneration, and impede recovery of a severe peripheral nerve injury.

Taxol (paclitaxel), a microtubule-binding compound, is one of the commonly used antineoplastic drugs for the treatment of solid tumours. Taxol binds along the length of microtubules and stabilizes them, which results in suppression of microtubule dynamics, leading to mitotic arrest and apoptosis in dividing cancer cells^1^,^2^. Neurons are also susceptible to taxol, and taxol exposure results in axonal degeneration^3^. Thus, taxol has serious side effects including peripheral neurotoxicity and myelosuppression. While administration of granulocyte colony-stimulating factor can counteract the neutropenia in most patients, there are no effective therapies to reduce or prevent the nerve damage, making neurotoxicity a significant dose-limiting side effect of taxol^4^,^5^,^6^. In the clinical condition, taxol typically induces sensory neuropathy, with the common symptoms being numbness, tingling, and burning pain. Sensory symptoms usually start symmetrically in the feet, but sometimes appear simultaneously in both the hands and feet. Most patients’ symptoms resolve within a few months after taxol treatment is stopped, but the abnormal sensory pain can occasionally become a chronic problem^5^.

Hypertrophic scarring and poor intrinsic axon growth capacity constitute major obstacles for neuron repair. These processes are tightly regulated by microtubule dynamics^7^. Microtubule stabilization promotes growth of central nervous system (CNS) axons of the raphespinal tract and leads to functional improvement. Thus, microtubule stabilization reduces fibrotic scarring and enhances the capacity of axons to grow^8^. It appears that the effects of microtubule stabilization by taxol on the neuron are dependent on the degree of stabilization. This is noted by recent studies describing taxol as a candidate treatment for promoting regeneration after central nervous injury. Recently, Hellal *et al*. found that after spinal cord injury in rats, taxol applied directly to the lesion promoted the formation of growth cones and regeneration of axons^8^. Moreover, an additional study of the mature CNS demonstrated that taxol promotes axon regeneration of the injured optic nerve when administered locally^9^,^10^.

An understanding of the mechanisms underlying taxol-induced neurotoxicity is important for developing therapeutics to prevent and alleviate the neuropathy. However, conflicting results on nerve injury/regeneration have been reported, and little information was found in the literature to examine the effect of taxol on the regeneration of injured peripheral nerves. To determine this effect with a standard accepted acute injury model of neuropathy^11^,^12^; we assessed the influence of taxol on a 10 mm sciatic nerve defect in rats, which was repaired with a silicone rubber nerve tube. After a recovery period, electrophysiology of the regenerated nerve was performed. To assess animal behaviour, thermal hyperalgesia tests, mechanical hyperalgesia tests, and motor coordination tests were studied. Retrograde labelling of dorsal root ganglions (DRGs) was performed to assess neuronal connectivity. We also assessed the number and morphology of the regenerated nerves, and macrophage infiltration in the distal nerve. In addition, we evaluated the regeneration of adrenergic axons in rat sciatic nerve using tyrosine hydroxylase (TH) immunostaining. Finally, expression levels of calcitonin gene-related peptide (CGRP) in the lumbar spinal cord were determined. These procedures were performed with the aim of elucidating the mechanisms underlying the observed effects of taxol treatment. Moreover, since the regenerative ability of nerves strongly relies on the regulation of growth factors and immune responses, the expression levels of nerve growth factor (NGF), platelet-derived growth factor (PDGF), and immunoregulatory factors, including tumour necrosis factor (TNF)α, granulocyte-macrophage colony-stimulating factor (GM-CSF), and CD68, were also investigated in the regenerated nerves.

## Results

### Regeneration across Gaps within Silicone Rubber Conduits

After an implantation time of 4 weeks, the silicone implants containing the regenerated cables were retrieved and examined. After trimming the fibrous tissue covering the nerve cuff, regenerated cables could be seen through the lumen of the cuff. Overall gross examination of the cuffs revealed successful regeneration in the control, Cremophor EL, taxol (2 mg/kg), and taxol (6 mg/kg) groups in 80%, 80%, 40%, and 30% of the rats, respectively. These results showed that cable formation within silicone tubes was significantly reduced in the taxol-treated rats.

### Electrophysiological Measurements

**Figure 1:**
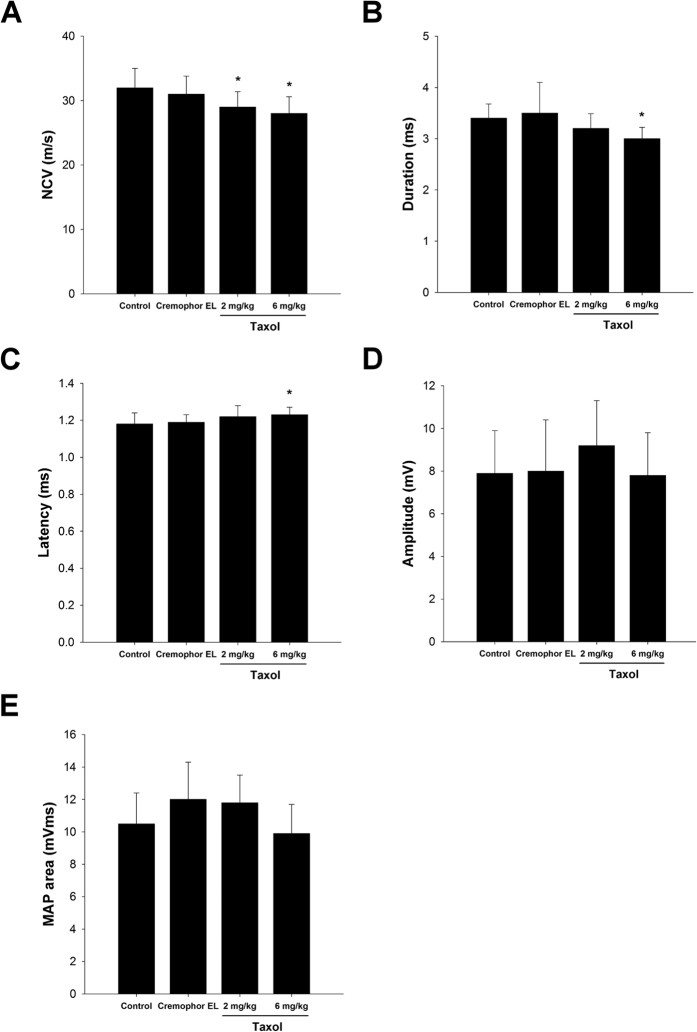
The nerve electrophysiological functions, including (**A**) nerve conductive velocity (NCV), (**B**) duration, (**C**) latency, (**D**) amplitude, and (**E**) muscle action potentials (MAP) area of the nerve post-injury are shown. The values represent means ± standard deviation (SD) for 10 rats for each group. *P < 0.05, compared to control and Cremophor EL groups.

Obvious excitability and conductivity were seen in all of the rats in the electrophysiological study, indicating that their regenerated nerve fibres had successfully reinnervated with the gastrocnemius muscle. However, the quantitative data demonstrated that nerve functions, including nerve conductive velocity (NCV) (Fig. 1A) and duration (Fig. 1B) were significantly impaired, and the latency (Fig. 1C) was significantly increased in 6 mg/kg taxol group as compared to the control or Cremophor EL groups (*P* < 0.05).

In comparison, amplitude (Fig. 1D) and MAP area (Fig. 1E) of the regenerated nerves did not differ significantly among the four groups, implying that the reinnervated muscles were in a seriously atrophic state even after 4 weeks of recovery.

### Thermal and Mechanical Hyperalgesia Tests

**Figure 2:**
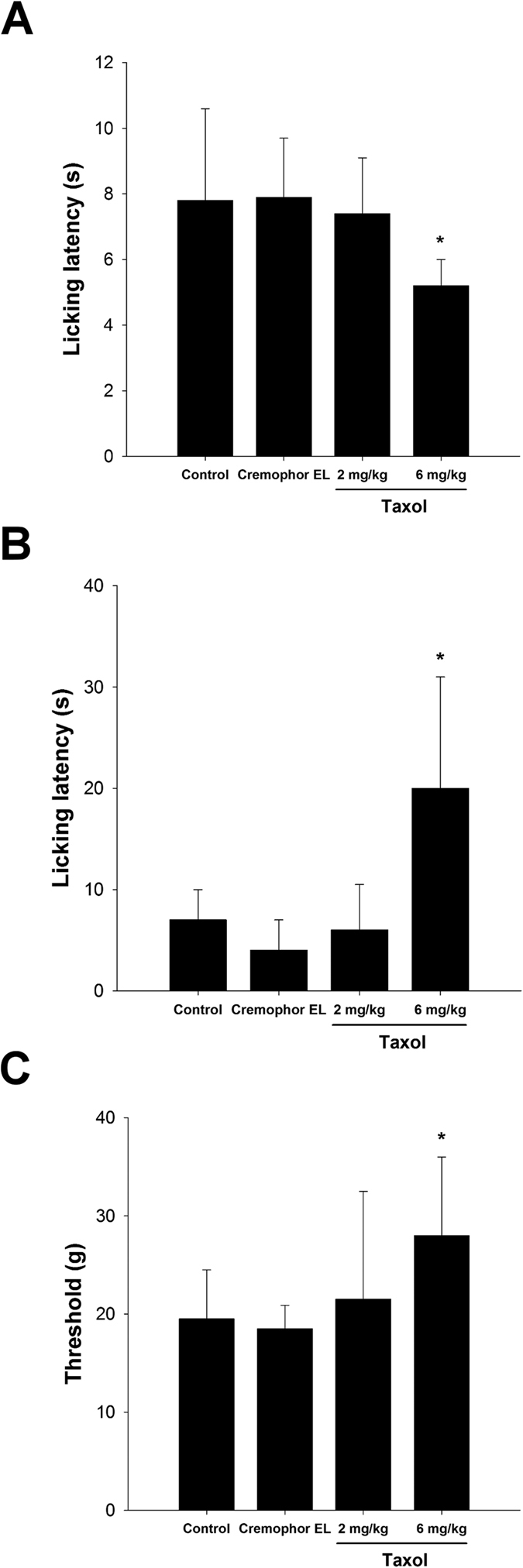
Effect of taxol on thermal and mechanical hyperalgesia tests. For the (**A**) radiant heat and (**B**) cold plate licking latency and (**C**) mechanical hyperalgesia test are shown. Licking is a rapid response to painful thermal stimuli that is a direct indicator of nociceptive threshold. Mechanical hyperalgesia was measured by testing the threshold force (g) of responses to stimulation with applications of electronic von Frey filaments. The values represent means ± standard deviation (SD) for 10 rats for each group. *P < 0.05, compared to control and Cremophor EL groups.

For the radiant heat test, the licking latencies for the taxol group (6 mg/kg) were significantly decreased as compared to the control and Cremophor EL groups (*P* < 0.05, Fig. 2A). By contrast, for the cold plate test, the licking latencies for the taxol group (6 mg/kg) were significantly increased as compared to the control and Cremophor EL groups (*P* < 0.05, Fig. 2B). In the mechanical hyperalgesia test, the threshold of pain for the rats in the taxol group (6 mg/kg) was significantly increased as compared to that of the control and Cremophor EL groups (*P* < 0.05, Fig. 2C). These results showed that taxol may reduce rats’ sensitivity to cold and mechanical stimulation.

### Motor Coordination Tests

**Figure 3:**
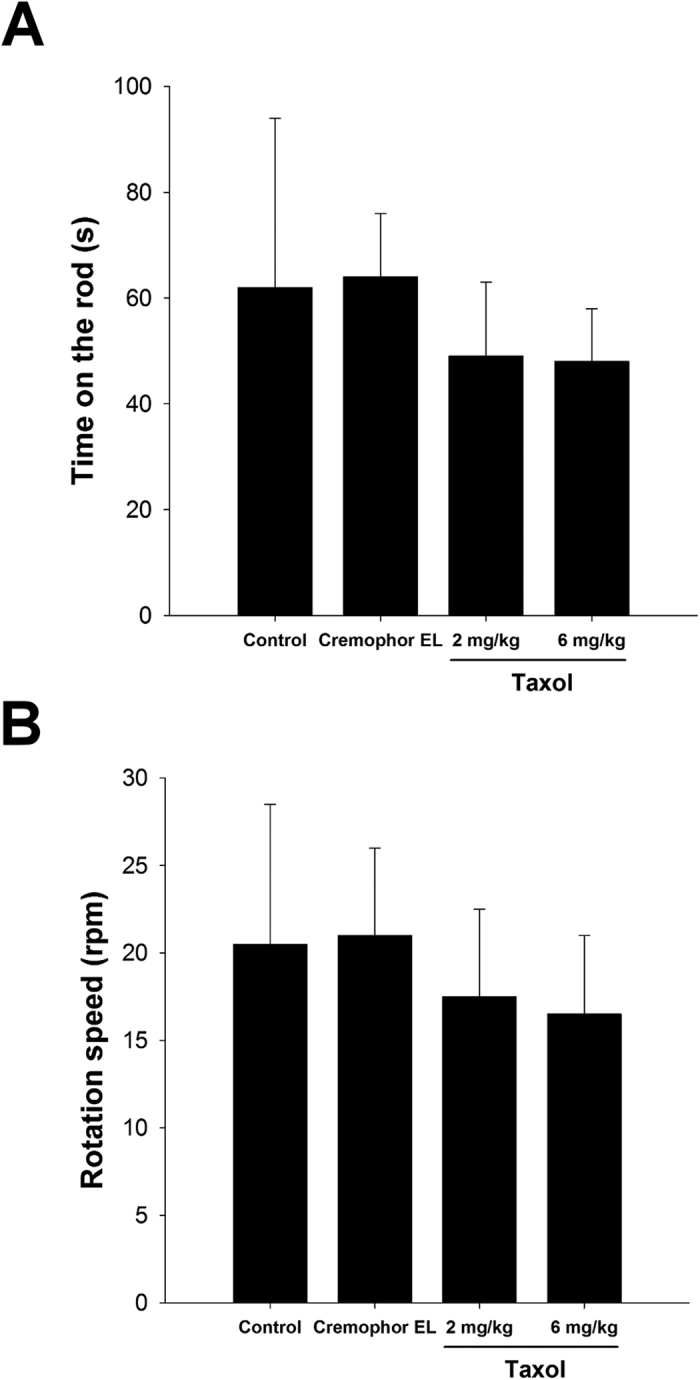
Effect of taxol on motor coordination tests. (**A**) Time-on-the-rod and (**B**) rod speed are shown. The values represent means ± standard deviation (SD) for 10 rats for each group.

For the motor coordination tests (time-on-the-rod and rod speed, Fig. 3A,B, respectively), although the motor coordination was slightly decreased in the taxol groups, there were no statistically significant differences found between all groups.

### Retrograde Labelling with Fluorogold

**Figure 4:**
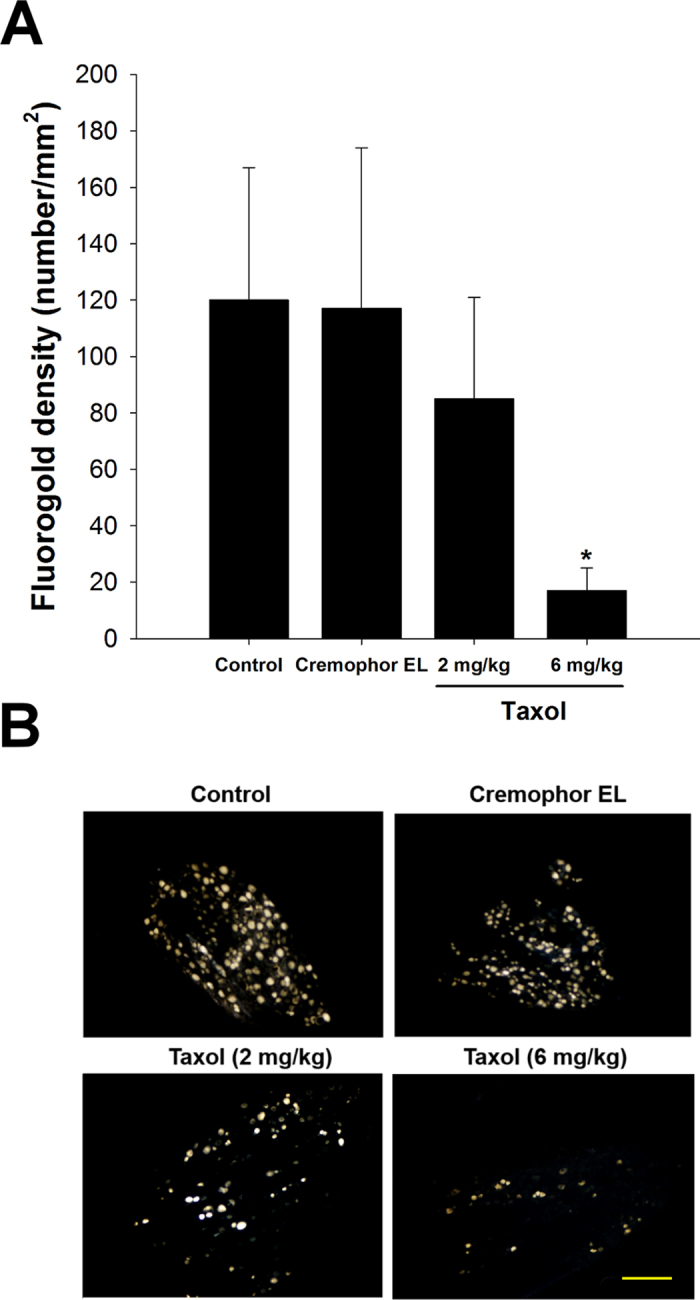
Retrograde labelling with Fluorogold. (**A**) The number of Fluorogold-labelled cell in DRGs was dramatically decreased in taxol-treated animals. (**B**) Representative images of the retrograde axonal tracing with Fluorogold. The values represent means ± standard deviation (SD) for 10 rats for each group. *P < 0.05, compared to control and Cremophor EL groups. Scale bar = 250 μm.

The Fluorogold-labelled cells in the cryostat section revealed that migrating axons had overcome the bridging nerve tissue in the tube and reached the DRG, indicating successful neuronal connectivity. It was found that the density of Fluorogold-labelling in the DRGs was significantly decreased in the taxol-treated groups, especially the group with 6 mg/kg of taxol as compared to the control and Cremophor EL groups (*P* < 0.05, Fig. 4A,B).

### Effect of Taxol on Maturity of Regenerated Nerves

**Figure 5:**
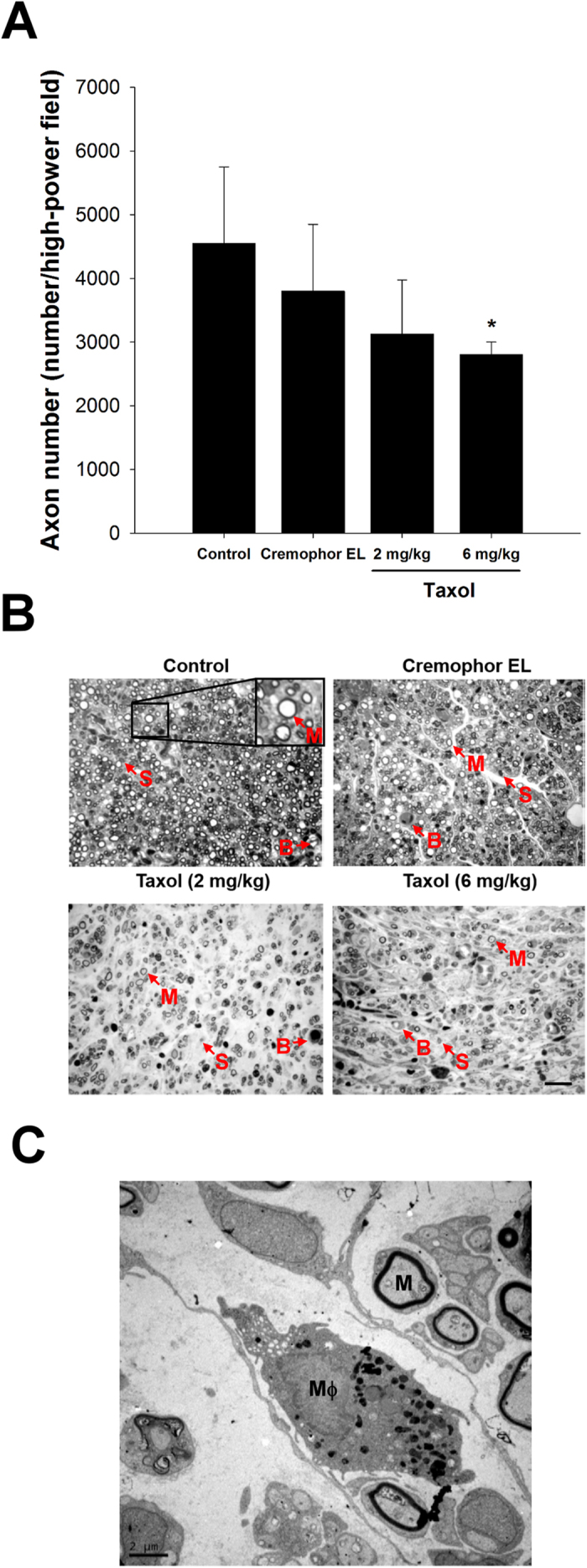
Effects of taxol on axon regeneration in rats after nerve injury surgery. (**A**) Quantitation of myelinated axonal counts in regenerated sciatic nerve cross-sections. (**B**) Representative histological micrographs of nerve tissue (M: myelinated axon; S: Schwann cell; B: blood vessel). (**C**) Ultrastructural analysis using electron microscopy to determine the macrophage (Mϕ) infiltration and remyelination in taxol-treated groups. The values represent means ± standard deviation (SD) for 10 rats for each group. *P < 0.05, compared to control and Cremophor EL groups. Scale bars = 20 μm and 2 μm for (**B**) and (**C**), respectively.

Dramatic differences were noted among the tissue cables treated with different concentrations of taxol. In the control and Cremophor EL groups, the successfully regenerated nerve cables had a relatively mature structure, in which a substantial portion of the endoneurial area was occupied by connective tissue with an abundance of myelinated axons. In comparison, the nerve cables in both taxol-treated groups had an immature structure, in which loosely distributed myelinated axons with a few blood vessels and Schwann cells were seen (Fig. 5A). Morphometric data also revealed that both groups of taxol-treated rats had a dramatic reduction in the number of myelinated axons as compared to the control. For rats treated with 6 mg/kg of taxol, this reduction was significant as compared to the saline control (*P* < 0.05, Fig. 5B).

Moreover, transmission electron microscopy (TEM) was used for ultrastructural analysis in taxol-treated groups (Fig. 5C). It was noted that a relatively large fraction of the core in the regenerated cable treated with taxol was filled with collagenous endoneurial connective tissue and several myelinated axons. In addition, it showed endoneurial macrophages in close proximity to Schwann cells containing myelinated axons, indicating the macrophage infiltration and remyelination.

### Macrophages Recruited in the Distal Nerve Ends

**Figure 6:**
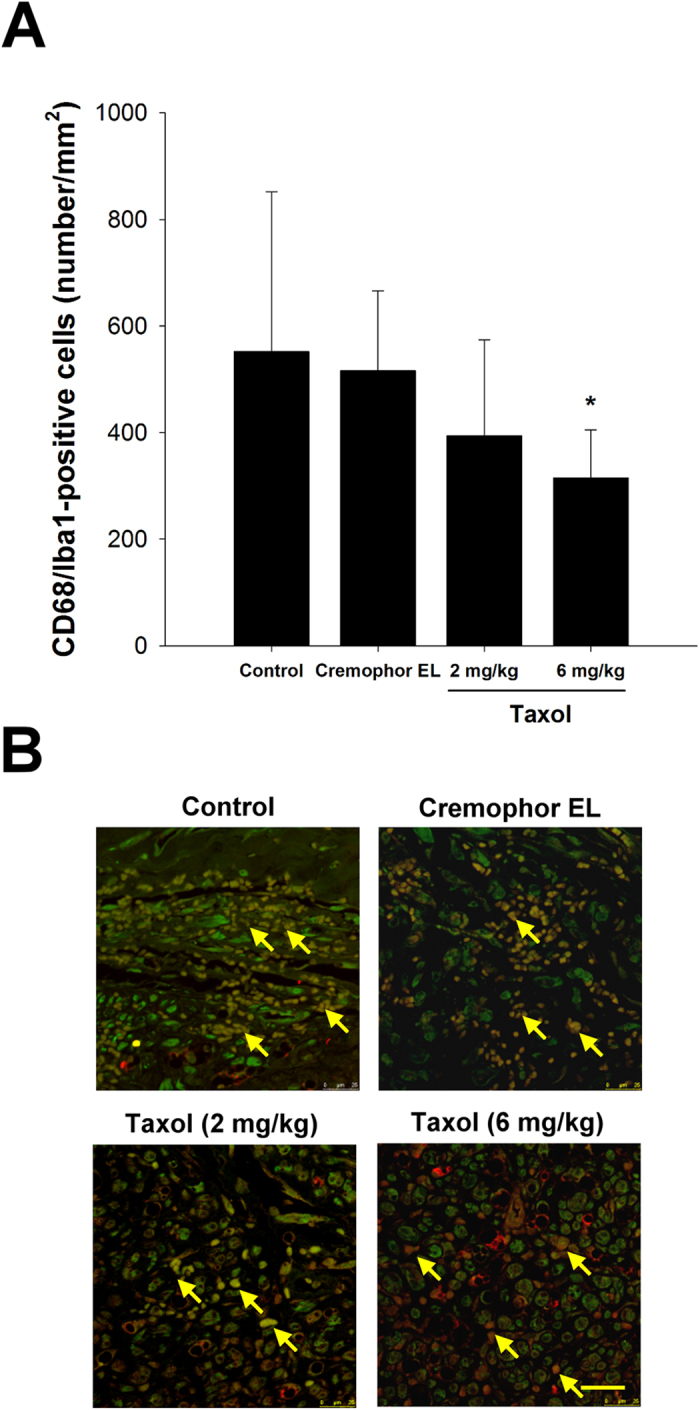
Effects of taxol on macrophage infiltration stained with CD68 and ionized calcium binding adaptor molecule 1 (Iba-1) in rats after nerve injury surgery. (**A**) Quantitation of macrophage infiltration density. (**B**) Representative photographs of CD68 and Iba-1 immunoreactivity on the macrophages (arrows). The values represent means ± standard deviation (SD) for 10 rats for each group. *P < 0.05, compared to control and Cremophor EL groups. Scale bar = 25 μm.

Post-injury macrophage infiltration revealed that continued damage and post-damage clearance as well as inflammation existed in the nerve injury lesion. It was noted that the density of stained macrophages was dramatically decreased in both groups of the taxol-treated animals, especially those treated with 6 mg/kg of taxol had significantly fewer macrophages as compared to the control and the Cremophor EL groups (Fig. 6A, *P* < 0.05). Figure 6B shows the expression of Iba1 and CD68 for regenerated nerves. The data with immunostaining for Iba1 and CD68 to quantify macrophage numbers reflect that the treatment of taxol may hinder the macrophage invasion into the injured sites.

### Regeneration of Adrenergic Axons in the Sciatic Nerve

**Figure 7:**
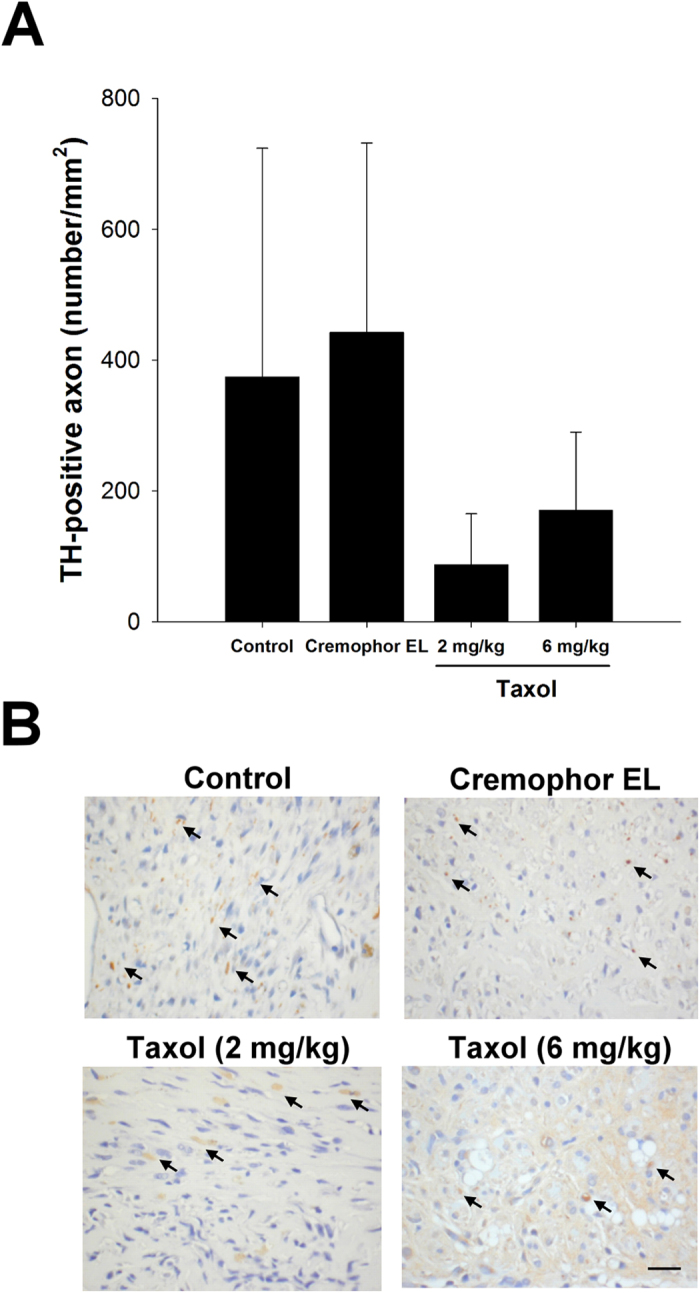
Effects of taxol on regeneration of adrenergic axons in rat sciatic nerve using tyrosine hydroxylase (TH) immunostaining. (**A**) Quantitation of TH-stained axon density in regenerated sciatic nerve cross-sections. (**B**) Representative histological micrographs of TH-stained axons (arrows). The values represent means ± standard deviation (SD). Scale bar = 20 μm.

We also evaluate the regeneration of adrenergic axons in rat sciatic nerve using TH immunostaining. As shown in Fig. 7A,B, TH-positive stained axons were illustrated as brown regions within the rat sciatic nerve. It was noted that the small sample size generated difficulty in comparing the effects of taxol on nerve regeneration statistically, because variations in axon counts. Nonetheless, the much fewer TH-positive staining of axons in taxol-treated animals as compared to the control and Cremophor EL groups still could provide strong evidence that the taxol was detrimental to the peripheral nerve regeneration.

### CGRP-immunereactivity in the Dorsal Horn

**Figure 8:**
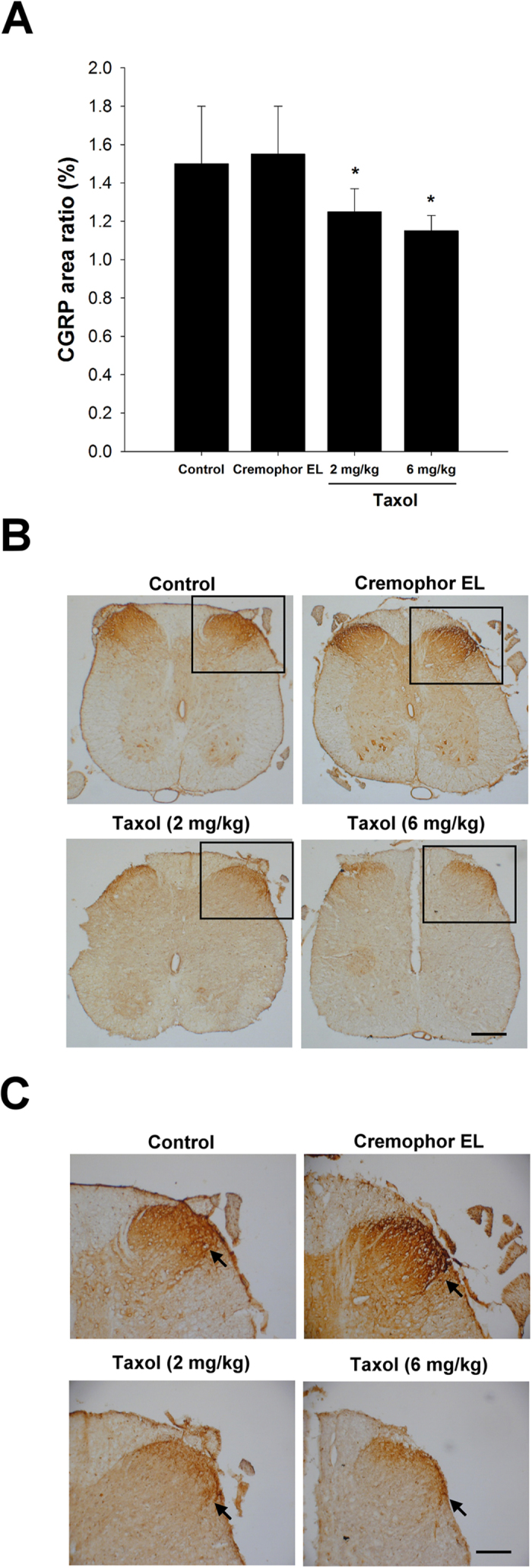
Effects of taxol on calcitonin gene-related peptide (CGRP) expression in rats after nerve injury surgery. (**A**) Quantitation for the ratio of CGRP expression area. (**B**) Representative histological micrographs of CGRP expression. (**C**) High magnification images show a visual difference in the statistics. The anatomic position of CGRP expression was separately accounted for the dorsal and ventral positions. The CGRP of dorsal horn (arrows) showed the highest expression of whole horizontal view of spinal cord. The values represent means ± standard deviation (SD) for 10 rats for each group. *P < 0.05, compared to control and Cremophor EL groups. Scale bars = 200 μm and 100 μm for (**B**) and (**C**), respectively.

The anatomic position of CGRP expression was measured separately for the dorsal and ventral positions. Immunohistochemical staining showed that CGRP-labelled fibres were seen in the area of lamina III-V and lamina I-II regions in the dorsal horn, ipsilateral to the injury in all of the rats. CGRP in the dorsal horn additionally showed the highest expression in the whole horizontal view of the spinal cord. Quantitative data showed that CGRP expression was significantly decreased (*P* < 0.05) in the taxol groups (2 and 6 mg/kg) as compared to the control and Cremophor EL groups, respectively (Fig. 8A,C).

### Gene Expression

**Figure 9:**
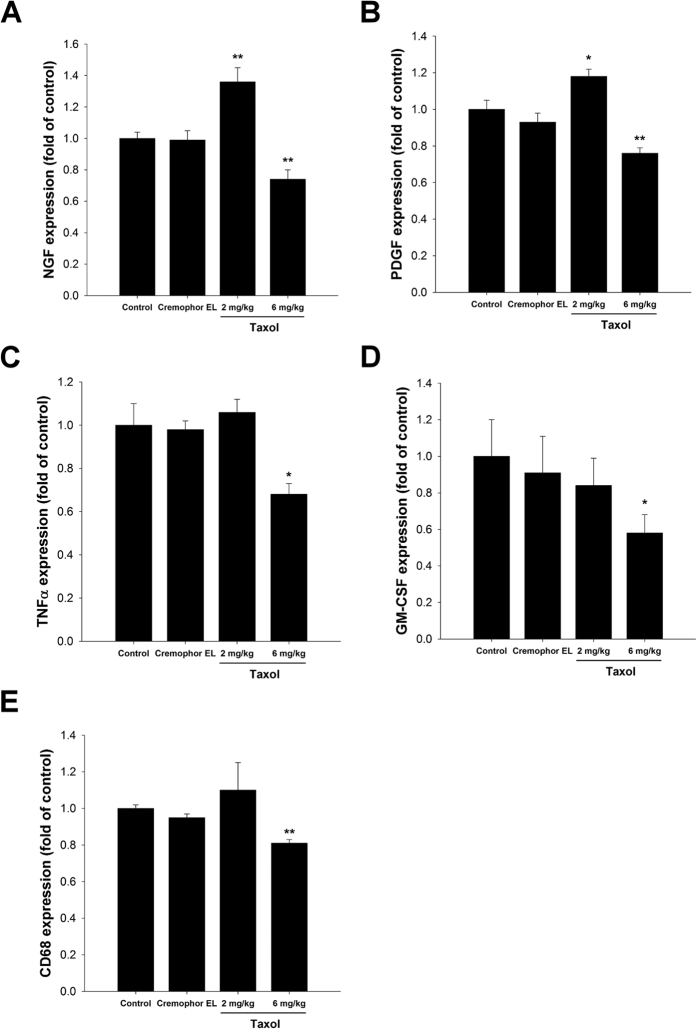
Effects of taxol on the mRNA expressions of (**A**) nerve growth factor (NGF), (**B**) platelet-derived growth factor (PDGF), (**C**) tumor necrosis factor (TNF)α, (**D**) granulocyte-macrophage colony-stimulating factor (GM-CSF), and (**E**) CD68 in the regenerated nerves of rats after nerve injury surgery. The values represent means ± standard deviation (SD) for 10 rats for each group. *P < 0.05; **P < 0.01, compared to control and Cremophor EL groups.

The regenerative ability of nerves strongly relies on the regulation of growth factors and immune responses. The expression levels of two growth factors, NGF and PDGF, were monitored by quantitative PCR analysis. The mRNA levels of both NGF (Fig. 9A) and PDGF (Fig. 9B) in regenerated nerves of the 2 mg/kg taxol-treated group were significantly higher than the control and Cremophor EL groups. However, mRNA levels of both growth factors were lower in the 6 mg/kg taxol-treated group. Immunoregulatory factors, including TNFα (Fig. 9C), GM-CSF (Fig. 9D), and CD68 (Fig. 9E), were also investigated in the regenerated nerves. The mRNA expression levels of all three factors in the nerves of the 6 mg/kg taxol-treated group were significantly lower than in other groups.

## Discussion

Taxol is an antineoplastic agent with a broad spectrum of activity. Taxol promotes microtubule assembly, and consequently, neurotoxicity is one of its side effects. Clinical use of taxol has led to peripheral neuropathy and this has been demonstrated to be dependent upon the dose administered, the duration of the infusion, and the schedule of administration. Vehicles such as Cremophor EL in the drug formulation have also been investigated for their potential to induce peripheral neuropathy^13^. A variety of neuroprotective agents has been tested in animal and clinical studies to prevent taxol neurotoxicity^13^.

Peripheral nerves are susceptible to a variety of injuries. Although the presence of nerve involvement in many circumstances may be clinically obvious, an appropriate therapeutic approach depends on more detailed information regarding the nature of the lesion. Electrophysiological measurements can provide precise information about the localization and severity of the nerve injury. These are particularly helpful when the experimental examination is limited by pain or poor effort on the part of the animals. They also provide information about prognosis, allowing one to reliably estimate the timing and extent of recovery^14^. Electromyography is also designed to investigate the amplitude and morphology of the electrical signal within skeletal muscle. The amplitude of the motor unit potential is dependent on the density of the muscle fibers attached to that one motor neuron. Motor conduction studies are performed by stimulating a motor nerve while recording the response from its target muscles. It is important to note that the electrical signal that is being recorded following motor nerve stimulation (muscle action potential; MAP) is actually generated by the muscle, and therefore it is quite large^14^.

In the present study, we investigated the effects of taxol on neuron regeneration in a rat sciatic nerve transection model. The electrophysiological data demonstrated that nerve functions, including NCV, duration, and latency were significantly impaired in the high-dose taxol group. The licking latencies and mechanical hyperalgesia for the high-dose taxol group at 6 mg/kg were significantly changed, suggesting taxol impairs the sensory function in peripheral neuropathy. Nevertheless, taxol did not affect the motor coordination, even motor-rod is a system that measures motor and sensory input as measures coordination on the rod. Fluorogold labelling in the DRGs, axon number, macrophage density, and CGRP expression were dramatically decreased in the high-dose taxol group. The results suggest that a high dose of taxol could significantly impair nerve regeneration in rats after acute peripheral nerve injury.

Unlike the results showing that taxol may reduce rats’ sensitivity to cold and mechanical stimulation, the licking latencies during the radiant heat test for the high-dose taxol group were significantly decreased. This could be caused by the involvement of transient receptor potential (TRP) ion channels which are responsive to temperature. Six TRP channels are proposed to be involved in thermosensation and are located in the sensory nerves and skin. For example, TRPV1, TRPV2, TRPV3, and TRPV4 channels have incompletely overlapping functions over a broad thermal range from warm to hot. Cool and cold temperatures are sensed by the TRPM8 and TRPA1 family members^15^. These channels need to be further investigated to identify previously unexplored mechanisms in taxol-modulated thermal hyperalgesia^16^.

Results in the present study differed from the reported effects of taxol on CNS regeneration. Taxol has been shown to facilitate axonal regeneration in the CNS after spinal cord injury by decreasing scar formation and enhancing intrinsic axonal growth. This is because taxol can moderately stabilize the microtubules which may counteract various cellular processes that prevent axon regeneration. Unlike the axons of the peripheral nervous system (PNS), which have the capacity to regenerate, central nervous axons form retraction bulbs at their tips after injury, and do not regrow. Normally, this retraction bulb has disorganized microtubules, but application of taxol after central nervous injury interferes with the formation of retraction bulbs. This causes microtubules in these axonal endings to become parallel and bundled, and no longer be disorganized^17^. Thus, taxol has been reported to offer a multi-targeted therapy for spinal cord injury^8^. All of these findings show that the dynamic properties of microtubules are critically important to axonal growth and regeneration^18^.

The inflammatory response that accompanies neural injury involves multiple cell types and effector molecules with either positive or negative effects. Inflammation is essential for normal regeneration in the peripheral nervous system, induction of inflammation within dorsal root ganglia, when combined with other treatments, enables peripheral sensory neurons to regenerate axons into the spinal cord. However, inflammation also has negative effects that impede recovery. In light of the importance of inflammation for neural repair, it is important to identify the specific cell types and molecules responsible for the positive and negative effects of inflammation and to develop treatments that tip the balance to favor repair^19^. Successful PNS regeneration relies on both injured axons and non-neuronal cells such as immune cells, especially the macrophages. Upon nerve injury, macrophages infiltrate the injury sites. Here, they not only contribute to Wallerian degeneration but are also polarized to an anti-inflammatory phenotype as a result of the influence of the local microenvironment, contributing to axonal regeneration^20^. Apart from their role in removing myelin debris from the degeneration process, the macrophages and their released cytokine interleukin-1β could also stimulate the secretion of various growth factors in dissected nerve segments, which could exert neurotrophic effects on regenerating nerve fibres^21^,^22^,^23^. In the present study, macrophage infiltration density was significantly decreased in the distal sciatic nerve after injury in the taxol-treated (2 and 6 mg/kg) groups. This may cause a delay in Wallerian degeneration accompanied with less secretion of neurotrophic factors, resulting in impaired nerve regeneration.

It has been reported that activation of accumulated macrophages in the DRG of taxol-treated rats could contribute to generation and development of the neuropathy^5^,^24^. In the studies of Peters *et al*., they found there was an increase in the number of CD68 positive activated macrophages within the DRG and peripheral nerve of rat after intravenous paclitaxel administration for 10 days. The difference between the above results may be related to differences in time periods and animal models^25^,^26^. In summary, the underlying mechanisms involved in moderating taxol could potentially regulate the inflammatory process, leading to modulation of the regenerative response.

CGRP is produced in both peripheral and central neurons and is a potent peptide vasodilator that can function in the transmission of pain^27^. In a spinal cord injury, CGRP is derived from motor neurons and plays a role in nerve regeneration after injury. Conversely, CGRP is derived from the DRG when synthesized in the dorsal horn of the spinal cord and may be involved in transmission of post-injury pain^27^. Previous studies showed that CGRP stimulates specific progenitor cells, which secrete an insulin-like growth factor, leading to regeneration^28^, and CGRP represses specific immune cells including T-lymphocytes^29^,^30^. Moreover, CGRP could be a biosignature for the surveillance of the basal and dorsal root CGRP enhancement, which might reflect the physiological status of the synaptic connections in the spinal dorsal horn^31^. In the present study, taxol significantly decreased CGRP expression in the dorsal horn of the treated rats. It is conceivable that suppressed CGRP expression in the spine may be attributable to the fact that taxol treatments provoked less injury-related signals retrogradely transported to neurons, and subsequently triggered fewer cells to synthesize and release CGRP.

Research examining the effects of taxol on sensory neuronal function has focused largely on *in vivo* studies using an animal model of taxol-induced neuropathy^5^,^32^,^33^,^34^,^35^. Various putative therapeutics (antidepressants, gabapentin, cyclooxygenase inhibitors, antioxidants, or immune suppressing agents)^36^,^37^,^38^,^39^,^40^,^41^ are moderately successful in the animal model, however, they fail in translation into the clinical condition^42^. Recently, Pittman *et al*. showed that a relatively low concentration of taxol augmented neurotransmitter release, whereas a high concentration reduced neurotransmitter release. Our quantitative PCR results on neuron-related growth factors are generally consistent with this study. In the low-dose taxol-treated group mRNA levels of both NGF and PDGF in regenerated nerves were significantly increased. In contrast, mRNA levels of both growth factors were lower in the high-dose taxol-treated group. The results are analogous to animal studies using systemic injection of taxol, which suggest that both sensitizing and desensitizing mechanisms may contribute to the clinical symptoms of neurotoxicity, dependent on the dose and on the experimental endpoints measured^42^.

Our study has certain limitations. First, behavioral testing was more thorough than electrophysiology; no gait analysis was shown and gait analysis in rodents in tricky but in rats seems to be an objective test for evaluating sensory polyneuropathy associated with chemical agents such as taxol. Unfortunately, the digits of the repaired foot in some of the rats were missing due to automutilation, making gait analysis extremely difficult. Future improvements in this project we plan to use bitter gels smearing on the repaired foot to avoid automutilation, which may help us to analyze the gait of locomotor function. Second, in the gene expression study of immunoregulatory factors, the data of CD68 expression from IHC and real-time PCR did not completely correlate. Quantification of these alterations of makers is essential for understanding the molecular mechanisms underlying this pathology. However, often protein levels and mRNA levels are regulated independently. Many proteomics/microarray comparisons demonstrate a low correlation between expression levels or changes between protein and mRNA. We may have the mRNA but not the protein due to different post-transcriptional regulation mechanisms in the different status^43^. Thus, it is important for enzyme-linked immunosorbent assay based estimation of these molecules of growth factors and inflammatory molecules. Moreover, CD68 is a pan macrophage antigen but is also an antigen for general endothelial activity for angiogenesis. Therefore, we used Iba1 to identify macrophages to rule out the fact that CD68 may have stained endothelial cells among macrophages. Since neovascularization is as significant as macrophage activity, we suggest that specimens double-stained by CD68 and Iba1 are required to clarify the actual roles of taxol in nerve regeneration.

In summary, our results provide evidence that peripheral nerve regeneration is slowed substantially in taxol-treated rats. A high dose of taxol may reduce nerve regeneration-related growth, expression of immunoregulatory factors, macrophage invasion into the injured sites, and CGRP expression in the spine, which could potentially deteriorate peripheral nerve regeneration processes.

## Methods

### Experimental Design and Surgical Procedures

This study was approved by the ethical committee for animal experiments of the China Medical University, Taichung, Taiwan. All experiments were performed in accordance with the use of Laboratory Animals (National Academy Press). After the adaptation period, the animals were anesthetized with an inhalational technique (AErrane; Baxter, Deerfield, IL), whose right sciatic nerves were severed into proximal and distal segments. The proximal stump was then secured with a single 9-Onylon suture through the epineurium and the outer wall of a silicone rubber chamber (1.47 mm inner diameterand 1.96 mm outer diameter; Helix Medical, Inc., Carpinteria, CA). The distal stump was secured into the other end of the chamber. Both the proximal and the distal stumps were secured to a depth of 1 mm into the chamber, leaving a 10-mm gap between the stumps. The muscle and skin were closed. All animals were housed in temperature (22 °C) and humidity (45%) controlled rooms with 12-hour light cycles. They had access to food and water *ad libitum*. Animals were divided into four groups. In group A (n = 10), rats were administrated with normal saline (i.p.) as the controls. In group B (n = 10), rats were administrated with Cremophor EL vehicle. Similarly, in groups C (n = 10) and D (n = 10), rats were administrated with 2 and 6 mg/kg of toxol in Cremophor EL solution (4 times in the 2-, 4-, 6-, and 8-day), respectively^5^,^44^.

### Animal Behaviour of Thermal and Mechanical Hyperalgesia

Four weeks after nerve repair, animal behaviour was tested before sacrifice. Thermal pain was measured with six applications using Hargreaves’ test IIT Canalgesiometer (IITC Life Sciences, SERIES8, Model 390 G)^45^. Both hot/cold-induced pains were measured using a hot/cold plate (Panlab, Harvarf Apparatus). Five minutes of animal behaviour were recorded using a digital camera and were analyzed offline using a personal computer^45^,^46^. Mechanical sensitivity was measured by testing the force of responses to stimulation with six applications of electronic von Frey filaments (North Coast Medical, Gilroy, CA, USA). All experiments were performed at room temperature and the stimuli were applied only when the animals were calm but not sleeping or grooming^47^.

### Radiant Heat Test

The radiant heat test was applied to assess the hyperalgesic behavior of the subjects. The test was performed using an apparatus (IITC Life Science Inc., USA) at 40 °C. The rats were placed in a transparent plastic box, and its window glass was an elevated flood. The rats must have adapted to the environment prior testing. The source of radiant heat was beneath the glass floor and focused at the hind paw plantar region. The timer of the test apparatus was on at the start of the simulation. The licking latency parameter was measured using a stopwatch. The paw was considered withdrawn when it was raised above the flood^47^.

### Cold Plate Test

The rats were placed onto a cold hot/cold plate apparatus (Panlab, Spain). Subjects were placed in a transparent plastic box that had a floor temperature of 4 °C^47^.

### von Frey Test

The von Frey test was performed to assess allodynia using a calibrated von Frey filament (IITC Life Science Inc., USA). Subjects were placed onto a metal mesh and stimulated by a tip of the filament at the plantar region. The filament gram counts were recorded when the stimulation caused the subject to withdraw its right hind paw^47^.

### Motor Coordination

Motor coordination was evaluated with an accelerating Rotamex Columbus instruments (Rotamex rotarod, Columbus Instruments, Columbus, OH). Accelerating rota-rod testing has been commonly used to assess motor performance associated with chemotherapy neurotoxicity^48^,^49^,^50^. Our initial setting was 6 rpm and the rotation was accelerated by 2.5 rpm every 10 s. The performance on the rod was measured in seconds from placement of the rat on the stationary rod to the time that it fell off following acceleration of the rod.

### Retrograde Labelling with Fluorogold

Fluorogold (Fluorochrome, Denver, CO, USA) was dissolved in distilled water to make a 2% suspension and stored at 4 °C in the dark. Immediately after the recording of muscle action potential, 1 μl and 2 μl of 2% Fluorogold solution were directly injected via a 10-μl Hamilton microsyringe into the common peroneal nerve and posterior tibial nerve. Following the injection, the site was wiped with a swab, flushed with sterile 0.9% saline, and the intraneural injection site closed with 10–0 nylon sutures. Then 5-mm distal to the injection site, the common peroneal nerve was ligated with 4–0 silk and 5-mm-long segments just distal to the ligation were harvested for histological assessment. The wound was then closed by 4–0 silk sutures. After allowing five days for retrograde transport, the animals were perfused transcardially with 200 ml of 0.9% saline, followed by 500 ml of ice-cold 4% paraformaldehyde in 0.1 M phosphate buffer. After perfusion, the L4 and L5 DRGs ipsilateral to the injury were dissected and removed, placed overnight in 4% paraformaldehyde in 0.1 M phosphate buffer for post-fixation and stored overnight in 30% phosphate-buffered sucrose solution. Frozen longitudinal sections of the spinal cord and DRGs, 40 μm thick, were made using a cryostat. After drying for 30 min, the sections were mounted and examined with an ultraviolet fluorescence microscope (Olympus ckx41, Center Valley, PA, USA). Retrogradely labelled neurones were then counted in the DRGs. The density of Fluorogold-labelled neurones was determined by dividing the neuron counts by the total DRG areas.

### Electrophysiological Techniques

After behaviour test, all animals were re-anesthetized and the sciatic nerve exposed. The nerve was given a supramaximal stimulus through a pair of needle electrodes placed directly on the sciatic nerve trunk, 5-mm proximal to the transection site. Latency, amplitude, and area of the evoked MAPs were recorded from the gastrocnemius muscle with microneedle electrodes linked to a computer (Biopac Systems, Inc., Goleta, California). The latency was measured from stimulus to the takeoff of the first negative deflection. The amplitude and the area under the MAP curve from the baseline to the maximal negative peak were calculated. The MAP was then used to calculate the NCV, which was carried out by placing the recording electrodes in the gastrocnemius muscles and stimulating the sciatic nerve proximally and distally to the silicone rubber conduit. The NCV was then calculated by dividing the distance between the stimulating sites by the difference in latency time.

### Histological Techniques

As abovementioned ptotocols, all of the rats were perfused transacrdially. The L4 spinal cord and the distal stump outside the nerve gap were quickly removed and post-fixed in the same fixative for 3 to 4 hours. Tissue samples were placed overnight in 30% sucrose for cry protection at 4 °C, followed by embedding in optimal cutting temperature solution. Samples were the kept at −20 °C until preparation of 18 μm sections was performed using a cryostat, with samples placed upon poly-L-lysine-coated slide. Immunohistochemistry of frozen sections was carried out using a two-step protocol according to the manufacturer’s instructions (Novolink Polymer Detection System, Novocastra). Briefly, frozen sections were required endogenous peroxidase activity was blocked with incubation of the slides in 0.3% H_2_O_2_, and nonspecific binding sites were blocked with Protein Block (RE7102; Novocastra). After serial incubation with rabbit- anti-CGRP polyclonal antibody 1:1000 (Calbiochem, Germany), Post Primary Block (RE7111; Novocastra), and secondary antibody (Novolink Polymer RE7112), the L4 spinal cord sections were developed in diaminobenzidine solution under a microscope and counterstained with haematoxylin. Similar protocols were applied in the sections from the distal stump except they were incubated with rabbit polyclonal anti-TH 1:500 (Millipore, UK). Sciatic nerve sections were taken from the middle regions of the regenerated nerve in the chamber. After the fixation, the nerve tissue was post-fixed in 0.5% osmium tetroxide, dehydrated, and embedded in Spurr’s resin. The tissue was then cut to 2-μm thickness by using a microtome (Leica EM UC6, Leica Biosystems, Mount Waverley, Australia) with a diamond knife, stained with toluidine blue, and observed under an optical microscope (Olympus IX70; Olympus Optical Co, Ltd, Tokyo, Japan). In addition, ultra-thin sections (70 nm) were made and stained with uranyl acetate and lead citrate, and were examined using a TEM at 100 kV (EM-UC7, Leica Microsystems, Wetzlar, Germany).

For immunofluorescent stain for macrophages, tissue sections were treated with 10% bovine serum albumin and 0.3% Triton X-100 for 1 h, and then the primary antibodies, dissolved in the same blocking solution, were applied at 4 °C overnight. The primary antibodies were mouse anti-CD68 (1:200; Serotec), and rabbit anti-Iba1 (1:100, Bioss, China). Tissue sections were washed thoroughly and then incubated with appropriate secondary antibodies tagged with Alexa Fluor 488 or 594 (1:500; Abcam) for 1 h at room temperature. The coverslips were mounted onto slides with aqueous-mount mounting medium (ScyTek, USA). The images were taken using a SP2/SP8X confocal microscope (Leica).

### Image Analysis

Tissue samples were observed under an optical microscope an image analyzer system (Image-Pro Lite; Media Cybernetics, Silver Spring, MD). CGRP-immunoreactivity in dorsal horn in the lumbar spinal cord was detected by immunohistochemistry as described previously^51^. The immuno-products were confirmed positive-labelled if their density level was over five times background levels. Under a 400x magnification, the ratio of area occupied by positive CGRP-immunereactivity in dorsal horn ipsilateral to the injury following neurorrhaphy relative to the lumbar spinal cord was measured. The number of neural components in each nerve section was also counted. As counting the myelinated axons, at least 30 to 50 percent of the sciatic nerve section area randomly selected from each nerve specimen at a magnification of 400x was observed. The axon counts were extrapolated by using the area algorithm to estimate the total number of axons for each nerve. Similarly, the total nerve areas were measured under the microscope at 40x. In addition, the density of macrophage was determined by dividing the macrophage counts by the total nerve areas.

### Quantitative PCR analysis

For rat, total RNA of tissue was isolated by MagNA Pure Compact RNA Isolation Kit (Roche, USA) and RNA samples were reverse-transcribed for 120 min at 37 °C with High Capacity cDNA Reverse Transcription Kit according to the standard protocol of the supplier (Applied Biosystems). The oligonucleotide primers used for this assay were corresponded to murine gene sequences (Table 1). All oligonucleotide primers were synthesized by Mission Biotech Co., LTD. in Taiwan. Quantitative PCR was performed by the condition: 10 min at 95 °C, and 40 cycles of 15 sec at 95 °C, 1 min at 60 °C using 2× Power SYBR Green PCR Master Mix (Applied Biosystems) and 200 nM of forward and reverse primers. Each assay was run on an Applied Biosystems 7300 Real-Time PCR system and expression fold-changes were derived using the comparative CT method. The mRNA of GAPDH served as the internal control for sample loading and mRNA integrity. The mRNA expression levels were calculated as a triplicate mean, with the results expressed as a percent relative to the control group compared to the treated group.

### Statistical Analyses

For the statistical analysis of immune-histochemically, morphometric, and electrophysiological measurements of regenerated nerves, data were collected by the same observer and expressed as mean ± standard deviation (SD), and comparisons between groups were made by the one-way analysis of variance using SAS 9.4 (SAS Institute Inc., Cary, NC, USA). The Tukey test was then used as a post hoc test for a multiple comparison. Statistical significance was set at *P* < 0.05.

## Additional Information

**How to cite this article**: Hsu, S.-T. *et al*. Effects of Taxol on Regeneration in a Rat Sciatic Nerve Transection Model. *Sci. Rep.* **7**, 42280; doi: 10.1038/srep42280 (2017).

**Publisher's note:** Springer Nature remains neutral with regard to jurisdictional claims in published maps and institutional affiliations.

## Figures and Tables

**Table 1:** Primer Sequence.

Primers	Sequences (5′-3′)
NGF-F	GTGGACCCCAAACTGTTTAAGAA
NGF-R	AGTCTAAATCCAGAGTGTCCGAAGA
PDGFα-F	AGGATGCCTTGGAGACAAACC
PDGFα-R	TCAATACTTCTCTTCCTGCGAATG
TNFα-F	GGCTGCCCCGACTACGT
TNFα-R	AGGGCAAGGGCTCTTGATG
GMCSF-F	ATGGCGCCTTGACCATGATA
GMCSF-R	ATGAAATCCTCAAAGGTGGTGACT
CD68-F	GCTGCAGGCTGCTCAGTTG
CD68-R	GGACCAGGCCAATGATGAGA
GAPDH-F	GCAAGTTCAACGGCACAG
GAPDH-R	CGCCAGTAGACTCCACGAC
