# Corrigendum: Effects of Taxol on Regeneration in a Rat Sciatic Nerve Transection Model

**DOI:** 10.1038/srep45421

**Published:** 2017-04-10

**Authors:** Shih-Tien Hsu, Chun-Hsu Yao, Yuan-Man Hsu, Jia-Horng Lin, Yung-Hsiang Chen, Yueh-Sheng Chen

Scientific Reports 7: Article number: 4228010.1038/srep42280; published online: 02 09 2017; updated: 04 10 2017

In this Article, Jia-Horng Lin is incorrectly listed as being affiliated with ‘Department of Fiber and Composite Materials, Feng Chia University, Taichung, Taiwan’. The correct affiliations are listed below:

Laboratory of Fiber Application and Manufacturing, Department of Fiber and Composite Materials, Feng Chia University, Taichung, Taiwan.

School of Chinese Medicine, China Medical University, Taichung, Taiwan.

Department of Fashion Design, Asia University, Taichung, Taiwan.

